# Photoinduced properties of anodized Ti alloys for biomaterial applications

**DOI:** 10.1038/s41598-023-41189-z

**Published:** 2023-08-25

**Authors:** N. Masahashi, M. Hatakeyama, Y. Mori, H. Kurishima, H. Inoue, T. Mokudai, K. Ohmura, T. Aizawa, S. Hanada

**Affiliations:** 1 Institute for Materials Research, Tohoku University, 2-1-1 Katahira, Aoba-ku, Sendai, 9808577 Japan; 2 Department of Orthopaedic Surgery, Graduate School of Medicine, Tohoku University, 2-1 Seiryo, Aoba-ku, Sendai, 9800872 Japan; 3 Department of Materials Science, Graduate School of Engineering, Osaka Metropolitan University, 1-1 Gakuen-cho, Naka-ku, Sakai, Osaka, 5998531 Japan

**Keywords:** Biomaterials, Biomedical engineering

## Abstract

The photocatalytic properties of anodic oxides on a newly developed TiNbSn and commonly used Ti6Al4V alloys as biomaterials were investigated. The alloys were anodized in an electrolyte of sodium tartrate acid with H_2_O_2_ at a high voltage and the mechanism of the photocatalytic and antiviral activities was studied. The anodized TiNbSn and Ti6Al4V exhibited highly crystallized rutile TiO_2_ and poorly crystallized anatase TiO_2_, respectively. X-ray photoelectron spectroscopy analysis revealed the presence of oxides of the alloying elements in addition to TiO_2_. The anodized TiNbSn exhibited higher activities than Ti6Al4V, and electron spin resonance spectra indicated that the number of hydroxyl radicals (⋅OH) generated from the anodized TiNbSn was higher than that from the anodized Ti6Al4V. The results can be explained by two possible mechanisms: the higher crystallinity of TiO_2_ on TiNbSn than that on the Ti6Al4V reduces the number of charge recombination sites and generates abundant ⋅OH; charge separation in the anodic oxide on TiNbSn due to the electronic band structure between TiO_2_ and the oxides of alloying elements enhances photo activities. The excellent photoinduced characteristics of the anodized TiNbSn are expected to contribute to the safe and reliable implant treatment.

## Introduction

Ti and its alloys are widely used as structural materials because of their high strength (490–1470 MPa for Ti alloys), corrosion resistance (less than 1 mm/year in 10% HCl), low density (4.51 g/cm^3^), and low Young’s modulus (108 GPa). Recently, their application in medical and dental devices has been increased considerably because of their high biocompatibility with tissues, in addition to the aforementioned properties. Biocompatibility is the ability of a material to function without a clinically important host response^1^. The biocompatibility of Ti originates from a several-nanometer-thick oxide layer present on its surface, which inhibits the redox reactions^2^. The oxide forms spontaneously on the surface upon exposure to air and is in thermodynamic equilibrium^3^, thereby functioning as a passivation layer responsible for the corrosion resistance and mitigating the release of metallic ions from the alloy^4^. If the oxide layer does not have sufficient resistant to wear and corrosion, it is easily disrupted by the interfacial shear stress, and bare Ti is exposed to corrosive body fluids, thereby leading to the elution of metal ions through the synergistic action of wear and corrosion^5^. Even if a new oxide layer develops on its surface after disruption, the rate of repassivation is very slow to prevent corrosion^6^, and the surface is damaged owing to the incessant stress^7^. Therefore, tribocorrosion-resistant and adhesive titanium oxides are required for biomaterials. The tetravalent form of TiO_2_ is well known for its biocompatibility, high chemical stability, and low toxicity^8^. The oxide is an n-type semiconductor photocatalyst that generates electrons and holes under ultraviolet (UV) light illumination, corresponding to a band gap energy (*E*_*g*_) of 3.2 and 3.0 eV for the anatase and rutile phases, respectively^9^. The generated charges react with water and oxygen in the atmosphere to yield reactive oxygen species (ROS) such as peroxides, superoxide, and hydroxyl radicals^10^. The ROS degrade surface-adsorbed toxic organic pollutants, bacteria, and viruses, unless charge carrier recombination occurs at lattice defects^11^. TiO_2_ has three crystalline forms^12^: anatase (tetragonal, a = b = 0.3782 nm, and c = 0.9502 nm), rutile (tetragonal, a = b = 0.4594 nm, and c = 0.2959 nm), and brookite (rhombohedral, a = 0.9185 nm, b = 0.5447 nm, and c = 0.5143 nm). The anatase and rutile exist at low and high temperatures, respectively, and brookite is rarely observed. It is reported that rutile exhibits lower photoactivity than anatase because of the high recombination rate of electron–hole pairs^13^ and the position of conduction band^14^. The lifetime of the photogenerated carriers of rutile is shorter than that of anatase^15^, owing to the direct band transition of charge carriers between the valence and conduction bands in the rutile, rather than the indirect band transition in the anatase^16^.

The electrochemical anodization forms titanium oxides (TiO_2_) on the surface of Ti and its alloys and strongly adhere to the substrate^17^. The oxides prepared at low voltages are amorphous^18^ and crystallize to anatase with increasing voltage, followed by a transition from the anatase to rutile^19^. Hatakeyama et al. reported that the formation of well-crystallized rutile oxide was promoted by using a high voltage during anodization on the TiNbSn alloy, and demonstrated the higher exfoliation strength of the rutile oxide on TiNbSn than that of the anatase oxide on Ti^20^. The formation of rutile is attributed to a spark discharge on the electrode surface because of the dielectric breakdown^21^. The energy density of the spark corresponding to a current density of 10^4^ A/cm^2^ (the surface temperature increases to ~ 8000 K)^22^ induces phase transformation in the metal substrate and anodic oxide^23^. In contrast, the anodization of pure Ti does not generate a spark discharge and forms a low-temperature stable anatase phase with low crystallinity^20^. An increase in the crystallinity of the oxide reduces the recombination sites, thereby improving photocatalytic activity^24^. Furthermore, the spark discharge accelerates the dissolution of the anodic oxide, leading to an increase of surface area and improvement of photoactivity. The authors reported that the anodized TiNbSn accompanied by spark discharge showed high surface area and crystallinity, in contrast, the anodized Ti exhibited without spark discharge low surface area and crystallinity^21^. The photoactivity of the anodized Ti was inferior to that of the anodized TiNbSn alloy, and the amount of generated hydroxyl radicals from the anodized Ti was lower than that from the anodized TiNbSn alloy. Herein, anodization of Ti alloys (Ti6Al4V and TiNbSn) was performed at a high voltage in a sodium tartrate electrolyte containing H_2_O_2_, which is similar to previous study, and the effect of alloying elements on photoinduced characteristics of the anodized alloys were investigated. H_2_O_2_ was added to electrolyte to promote electrochemical oxidation reaction because H_2_O_2_ is a strong oxidizing agent. Anodized Ti and TiNbSn alloy prepared in the electrolyte containing H_2_O_2_ exhibited higher crystallinity and better photoactivity than those without H_2_O_2_^21^. The Ti6Al4V alloy with hcp α and bcc β phases is commonly used in medical applications under load-bearing conditions owing to their high specific strength, fatigue strength, and corrosion resistance^25^. However, vanadium toxicity is a significant concern in these applications because the vanadium ions released from implants may cause implant failure by impairing normal bone deposition and inducing an inflammatory response^26^. In contrast, the near-β TiNbSn alloy comprises non-cytotoxic elements^27^ and exhibits a lower Young’s modulus (40–50 GPa)^28^ than the Ti6Al4V alloy (112 GPa)^29^. The low Young’s modulus suppresses stress shielding caused by the significant difference in the Young’s moduli between the hip prosthesis and cortical bone (10–30 GPa)^30^. Integrating photocatalytic properties, such as antibacterial function, to the TiNbSn alloy will expand the application of this alloy to medical implants. In this study, the photocatalytic properties of the anodized TiNbSn alloy were investigated in comparison with those of Ti6Al4V alloy, and the mechanism of photoinduced characteristics was discussed.

## Methods

### Preparation of anodized Ti alloys

A Ti6Al4V plate (Ti—6.62 wt.% Al—4.48 wt.% V) was supplied by Titan Meister (Akita, Japan). The TiNbSn alloy (Ti—34.52 wt.% Nb—4.11 wt.% Sn) was prepared by thermo-mechanical processing reported in an earlier paper^20^. The Ti alloys with dimensions of 25 mm × 25 mm × 1 mm, 10 mm × 20 mm × 1 mm, and 10 mm × 10 mm × 1 mm were galvanostatically anodized in the electrolyte of 50 mM sodium tartrate with 0.7 M H_2_O_2_ at a constant current density of 50 mA/cm^2^ for 0.5 h using a DC power supply (Matsusada Precision, PRK 500-3.2, Japan). Sodium tartrate is soluble in water (dissociation constant Pk_a_ is 4.34) and is approved as a food additive in EU (E337). H_2_O_2_ was added to electrolyte to promote electrochemical oxidation reaction because H_2_O_2_ is a strong oxidant to promote anodic reaction^21^. A 100 mesh Pt electrode with a size of 50 × 50 mm^2^ was used as the cathode. Anodized alloy plates were rinsed with distilled water, dried at 293 K, and annealed in air for 5 h at 723 K.

### Microstructural observation and surface analysis

The microstructures of the anodized alloys were observed using field-emission electron probe microanalysis (EPMA, JXA-8530F, JEOL, Japan), laser microscopy (LM, VK-X 150, Keyence, Japan), and transmission electron microscopy (TEM, EM-002B, Topcon, Japan). A thin film specimen for TEM observation was prepared using an ion slicer (EM-09100IS, JEOL, Japan). The crystallographic structures were determined using X-ray diffraction (XRD, X’Pert diffractometer, PANalytical, Netherlands) with a Cu-Kα radiation source operated at a scan rate of 1°/min, a sample possessing a thin-film geometry, and a glancing angle of 0.5°. X-ray photoelectron spectroscopy (XPS, Kratos AXIS-Ultra DLD, Shimadzu, Japan) was performed with monochromatic Al-Kα radiation at a base pressure of 3.0 × 10^−7^ Pa. The full width at half maximum (FWHM) intensity of the Ag 3d_5/2_ peak was 0.73 eV, and the base pressure of the spectrometer was 6.5 × 10^–8^ Pa. Binding energies were calibrated by the C1s peak from hydrocarbon at 284.8 eV. The spectral background was subtracted using Shirley's method. The spectra were analyzed using Casa XPS software (https://www.casaxps.com).

### Photocatalytic characteristic evaluation

The anodized Ti alloy plate (10 mm × 20 mm × 1 mm) was used for photocatalytic characteristic evaluation. The absorption spectra were measured using a UV–visible (vis) spectrophotometer (V-550, Jasco, Japan). The photocatalytic activity was evaluated by methylene blue (MB) bleaching tests according to the evaluation method approved by the Japanese Industrial Standards (JIS), R 1702. The anodized Ti alloy was placed in an optical quartz cell (10 mm × 10 mm × 44 mm) containing 2 ml of 3.19 ppm MB aqueous solution until the concentration of MB became constant to avoid the effect of MB adsorption on the photoactivity. A UV lamp (Xenon lamp MAX-350, Asahi Spectra, Japan) supplied UV light in the range of 260–390 nm using cut-off filter, and the intensity of the irradiated light was 1.0 mW/cm^2^ at the surface of the cell. The experimental data were fitted to a pseudo-first-order kinetic model based on the Langmuir–Hinshelwood kinetic model^31^:

1 $$\mathrm{r}=\frac{\mathrm{d}C}{\mathrm{d}t}=\frac{\mathrm{kK}C}{1+\mathrm{K}C}\approx {\mathrm{k}}_{\mathrm{app}}C $$

where r is the degradation rate of MB, *C* is the absorbance of MB at 664 nm, *t* is the illumination time, k is the reaction rate constant, and K is the reactant adsorption coefficient. When the initial concentration of MB is low, Eq. (1) can be written as an apparent first-order equation using the apparent rate constant k_app_. The antimicrobial activity was assessed following the protocol outlined in the International Organization for Standardization ISO 27447:2019 (JIS R 1702:2012). Gram-positive cocci, specifically methicillin-susceptible *Staphylococcus aureus* (MSSA; NBRC12732) was utilized for the antimicrobial experiments. Bacterium was incubated on nutrient agar medium (Difco nutrient agar, Becton Dickinson, Lake Franklin, USA) at a temperature of 35 °C for a duration of 36–43.5 h. Subsequently, the incubated bacteria were prepared in a nutrient broth medium (Nutrient broth, Eiken Chemical, Tokyo, Japan) with a concentration of 1:500, resulting in a bacterial count of 5.3 × 10^6^ cells/ml. This prepared solution was employed for the antimicrobial analysis. Glass plates were employed as negative controls, following a methodology described in a previous study^32^. For the ISO 27447 antimicrobial assay, three anodized TiNbSn and Ti6Al4V alloys and three glass plates were employed. The test bacterial solution (37.5 μL, 2 × 10^5^ cells) was inoculated onto both the anodized alloy samples and the reference glass plates, with the bacterial solution and specimen adhering to a sterile polyethylene film (VF-10, Kokuyo, Japan). Glass plates with a thickness of 1.1 mm (TEMPAX, Schott, Mainz, Germany) were placed on top of the Petri dish to prevent desiccation. The bacteria were then incubated at 25 °C for 8 h under UV illumination (FL 20S Bl-B 20 W, Nippon Electric Company, Japan) at a wavelength of 352 nm. The intensity of the UV light transmitted through the film and glass plate was adjusted to 0.21 mW/cm^2^. Additionally, bacteria on each sample were incubated at 25 °C for 8 h in the dark as a control group. Following UV illumination, the specimens and films were transferred to plastic bottles and washed with 20 mL of soybean casein digestion broth (SCDLP broth, Eiken Chemical, Japan), which contained lectin and polysorbate, to eliminate the test bacterial solution. A 1:10 dilution of the bacteria-washing solution was prepared by diluting 100 μL of the bacteria-washing SCDLP medium solution with 900 μL of saline solution. Subsequently, 100 µL of the bacteria-washing solution and 1:10 diluted washing solution were added to the nutrient agar medium and incubated accordingly. After an incubation period of 120 h at 35 °C on the nutrient agar medium, the number of colonies was counted to determine the viable count. The antibacterial activity value (R_L_) and the effect of UV light irradiation (ΔR) were calculated using the following equation:

2 $${\mathrm{R}}_{\mathrm{L}}={\mathrm{log}}_{10}\left(\frac{{G}_{L}}{{T}_{L}}\right) \Delta \mathrm{R}={\mathrm{log}}_{10}\left(\frac{{G}_{L}}{{T}_{L}}\right)-{\mathrm{log}}_{10}\left(\frac{{G}_{D}}{{T}_{D}}\right) $$

where *T*_*L*_ and *T*_*D*_ are the average viable bacterial counts on the three alloy and glass plates (25 mm × 25 mm × 1 mm) after UV illumination and after storage in the dark, respectively, for 8 h. *G*_*L*_ and *G*_*D*_ are the average viable bacterial counts on the three glass plates after UV illumination and after storage in the dark, respectively, for 8 h. The antimicrobial activity was defined as 2.0 or higher following the ISO 27447 (JIS R 1702). When no viable bacteria were observed on the anodized TiNbSn alloy group, the viable bacterial count (*T*_*L*_) was set to 10.

### Hydroxyl radical measurement

The number of hydroxyl radical (⋅OH) generated under UV illumination at a wavelength of 365 nm was measured using X-band electron spin resonance (ESR, JEOL, JES-FES-100, Japan). The surface of the anodized Ti alloy plate, with dimensions of 10 mm × 10 mm × 1 mm, was illuminated at an intensity of 1 mW/cm^2^ for 5 and 15 min using a UV lamp (SLUV-4; As One Corp., Japan). A spin-trapping agent [5,5-dimethyl-1-pyrroline-N-oxide (DMPO)] was used to measure the average value of ･OH in the three plates. The half-lives of the DMPO and its derivative 5-(diethoxy phosphoryl)-5-methyl1-pyrroline-1-oxide (DEPMPO), which forms a stable adduct with O^2−^, are 1 h and 15 min, respectively^33^. Therefore, the UV irradiation was set to a maximum of 15 min in this study. The test procedure is described in literature^34^.

## Results

**Figure 1:**
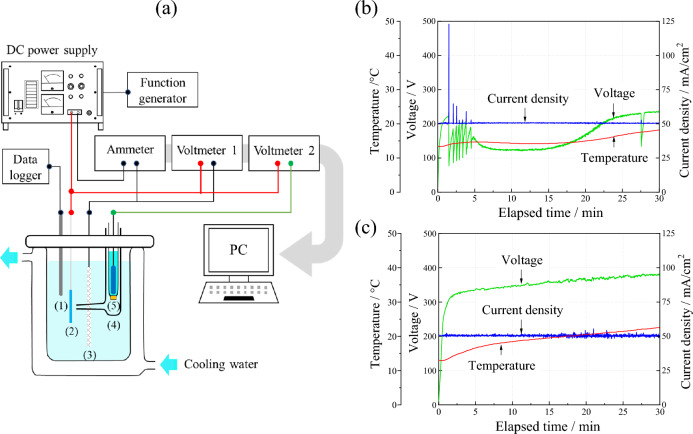
(**a**) Experimental setup for anodization; (1) thermocouple, (2) Ti alloy anode, (3) Pt cathode, (4) Luggin capillary, and (5) Ag/AgCl reference electrode. Variation in the voltage, current density, and temperature over time during anodization on (**b**) Ti6Al4V and (**c**) TiNbSn.

The electrochemical behavior during anodization was monitored by the electrode voltage, electrolyte voltage, and electrolytic current using a three-electrode configuration comprising a counter electrode and a Luggin capillary, with Ag/AgCl as the reference electrode. Figure 1 shows the schematic of the anodization experimental setup (Fig. 1a), and the variations in the electrode voltage and electrolyte temperature over time during the anodization of Ti6Al4V (Fig. 1b) and TiNbSn (Fig. 1c), respectively. In the TiNbSn substrate, the voltage increases gradually to 380 V over time. In the Ti6Al4V substrate, voltage increases gradually to 220 V, followed by a steep drop to 85 V after 5 min, thereafter, it vigorously fluctuates and eventually approaches to 230 V. The impedance of the anodic oxides can be calculated by the potential at the end of electrolysis, and the impedance of the anodized Ti6Al4V is estimated to be about 1/11th of the anodized TiNbSn. This suggests that chemical reaction of anodized Ti6Al4V is relatively active compared to anodized TiNbSn. The oscillations of potential and current in Fig. 1b is considered to be caused by the breakdown and restoration of the anodic oxides, which does not occur in the anodization in the electrolyte without H_2_O_2_ (not present in this paper). This indicates that the addition of H_2_O_2_ to the electrolyte increases oxygen or hydroxyl ions produced by oxidation of water molecules, promoting oxidation reaction. The electrolyte temperature of the TiNbSn and Ti6Al4V substrates gradually reaches to 22 and 18 degrees in Celsius, respectively, after 30 min of anodization. A few minutes after the start of anodization, a spark was observed on the surface of the TiNbSn electrode, whereas no sparks were observed on the surface of the Ti6Al4V electrode.

**Figure 2:**
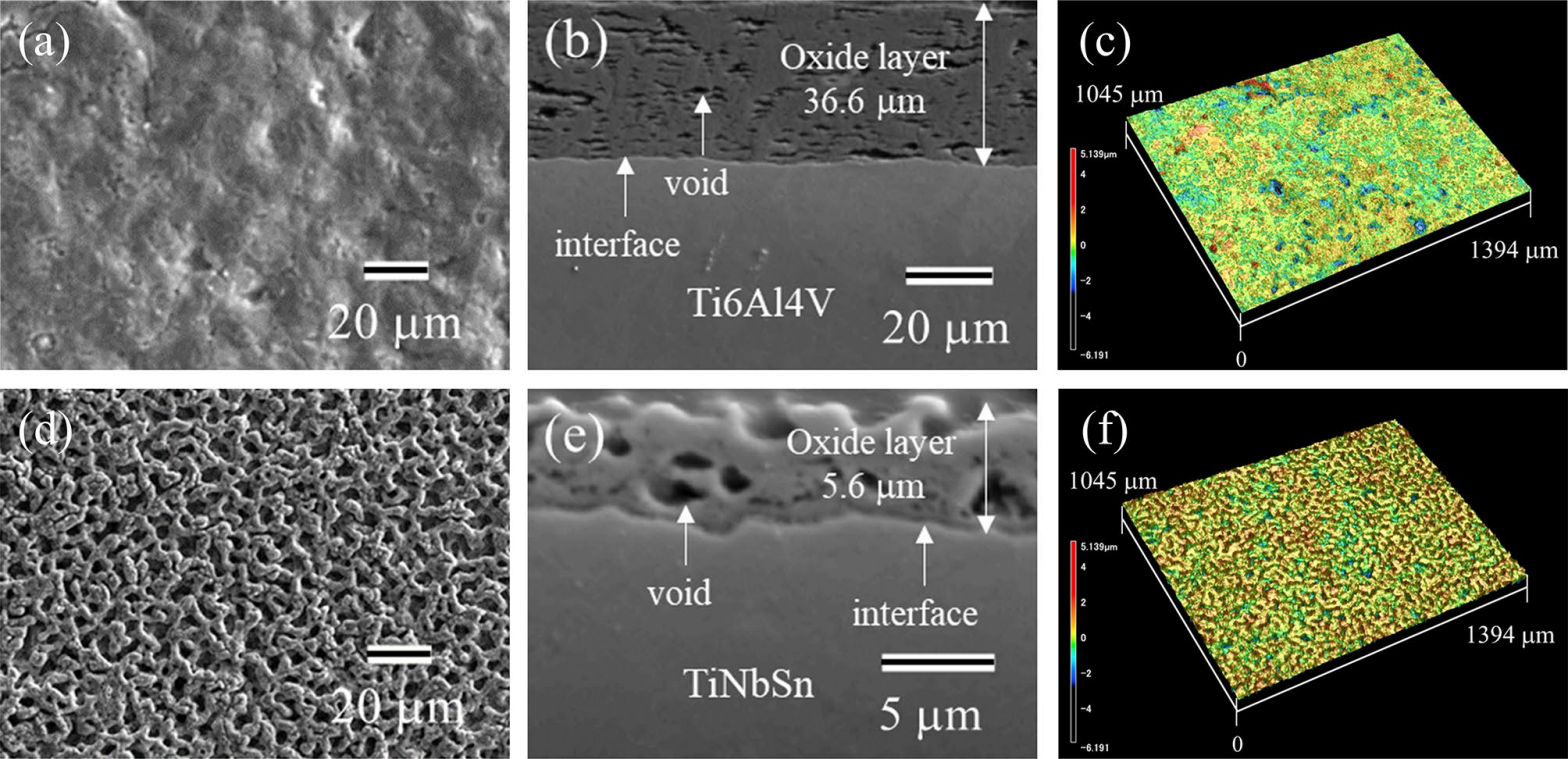
(**a,b,d,e**) SEM and (**c,f**) LM images of the anodic oxides on (**a–c**) Ti6Al4V and (**d–f**) TiNbSn; (**a,d**) plan-view, (**b,e**) cross-sectional, and (**c,f**) three-dimensional images of the oxide.

Figure 2 shows the scanning electron microscopy (SEM) (Fig. 2a, b, d, e) and LM (Fig. 2c, f) images of the anodic oxides on Ti6Al4V (Fig. 2a–c) and TiNbSn (Fig. 2d–f) surfaces. The plan-view SEM images (Fig. 2a, d) and LM images demonstrate a glassy and porous microstructure of the oxide on Ti6Al4V and TiNbSn surfaces, respectively. The cross-sectional images of the oxide on Ti6Al4V (Fig. 2b) shows a layered structure containing high-density pores parallel to the interface, whereas that on TiNbSn (Fig. 2e) shows homogeneously distributed pores. The thickness, surface roughness, and surface area ratio of the anodic oxides for the anodic oxides on Ti6Al4V and TiNbSn were 36.6 μm and 5.0 μm, 2.87 μm and 2.43 μm, and 4.13 and 3.26, respectively. Here, the surface area ratio is defined as a ratio of the measured surface area to the projected area. The oxide thickness of the oxide on Ti6Al4V is larger than that on TiNbSn. The anodic oxide on TiNbSn exhibits higher roughness and surface area ratio than the oxide on Ti6Al4V, probably due to dissolution of the oxide under a high voltage application.

**Figure 3:**
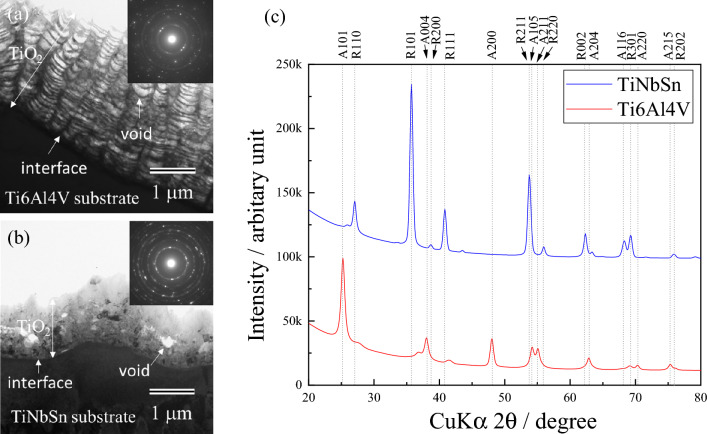
Cross-sectional TEM images of the anodic oxides on (**a**) Ti6Al4V and (**b**) TiNbSn, and (**c**) thin film XRD scan profiles of the anodized Ti alloys.

Figure 3 demonstrates the cross-sectional transmission electron microscopy (TEM) images of the anodic oxide on Ti6Al4V (Fig. 3a) and TiNbSn (Fig. 3b), and the XRD profiles of the anodic oxides (Fig. 3c). A layered oxide structure with alternating low and high-density pores is observed in the anodic oxide on Ti6Al4V (Fig. 3a). Conversely, homogeneously distributed pores appear in the oxide on the TiNbSn alloy (Fig. 3b). The selected area diffraction patterns of the oxides (insets in Fig. 3a, b) are indexed to the anatase and rutile phases, respectively. The XRD profiles (Fig. 3c) revealed that anatase and rutile TiO_2_ are observed as the dominant oxides on Ti6Al4V and TiNbSn, respectively. The rutile fraction was estimated the following equation^35^.

3 $${\mathrm{f}}_{\mathrm{rutile}}=0.679\times \left(\frac{{I}_{rutile}}{{I}_{rutile}+{I}_{anatase}}\right)+0.312\times {\left(\frac{{I}_{rutile}}{{I}_{rutile}+{I}_{anatase}}\right)}^{2} $$

where *I*_*anatase*_ and *I*_*rutile*_ are the peak areas of the anatase and rutile reflections, respectively. The crystallite size (L) and inhomogeneous lattice strain (ε) of the oxide were calculated from the full width at half maximum (FWHM) of the oxide peak using the Williamson Hall equation^36^:

4 $$\upbeta \cdot \mathrm{cos}\theta =\mathrm{K}\cdot \frac{\uplambda }{\mathrm{L}}+2\upvarepsilon \cdot \mathrm{sin}\theta $$

where L is expressed in nm, λ is the X-ray wavelength, β is the FWHM of the signal in radians, K is the shape factor (0.89, assuming that the crystallite is spherical); ε is the lattice strain, and *θ* is the Bragg angle. The calculated rutile fraction, ε, and L of the anodic oxide on Ti6Al4V and TiNbSn are 0.17 and 0.98, 0.22 and 0.02, and 8.8 nm and 15.8 nm, respectively. The ε value of the rutile oxide on TiNbSn is much lower than that of the anatase oxide on Ti6Al4V, suggesting that the oxides on the Ti6Al4V alloy accumulates high strain. The L of the rutile oxide on TiNbSn is larger than that of the anatase oxide on Ti6Al4V, owing to the coarse crystallites induced by the spark discharge during the anodization of the TiNbSn substrate. This spark discharge observed in the anodization of TiNbSn electrode induced the formation of rutile TiO_2_.

**Figure 4:**
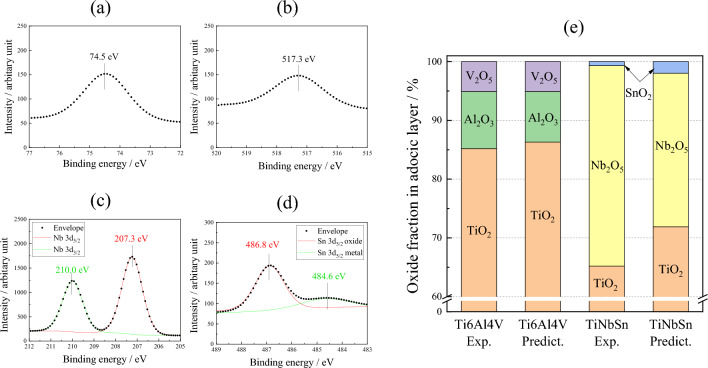
XPS spectra of (**a**) Al 2p, (**b**) V 2p from the anodic oxide on Ti6Al4V, and (**c**) Nb 3d, and (**d**) Sn 3d from the anodic oxide on TiNbSn. (**e**) Molar fractions of detected and predicted oxides on Ti alloys.

XPS analysis verified a formation of TiO_2_ on Ti alloys from the Ti 2p spectra at approximately 464.5 and 458.8 eV correspond to the Ti^4+^ 2p_1/2_ and Ti^4+^ 2p_3/2_ contributions of TiO_2_, respectively. Moreover, Ti^3+^ functioning as recombination sites for the charge carriers^37^ are not detected. Figure 4 shows the XPS spectra of Al 2p (a), and V 2p (b) from the anodic oxide on Ti6Al4V, and Nb 3d (c), and Sn 3d (d) from the anodic oxide on TiNbSn. The Al 2p peak (Fig. 4a) at 74.5 eV corresponds to Al_2_O_3_, and the V 2p peak (Fig. 4b) at 517.3 eV corresponds to V_2_O_5_. The Nb 3d spectrum (Fig. 4c) shows dual peaks at 207.3 and 210.0 eV, representing Nb^5+^ 3d_5/2_ and Nb^5+^ 3d_3/2_ of Nb_2_O_5_, respectively. The Sn 3d spectrum (Fig. 4d) at 486.8 eV corresponds to SnO_2_. The fractions of the species detected in the anodic oxides on Ti6Al4V and TiNbSn were calculated via semi-quantitative analysis assuming that the surface of the anodic oxides comprises metal oxides as shown in Fig. 4e. The molar fraction of TiO_2_ in the anodic oxides on the Ti6Al4V surface is higher than that in the anodic oxides on the TiNbSn surface, owing to the higher concentration of Ti in Ti6Al4V than that in TiNbSn. The calculated fractions of Al_2_O_3_ and Nb_2_O_5_ in the anodized Ti6Al4V and TiNbSn, are higher than the fractions predicted from the nominal composition of Ti alloys.

**Figure 5:**
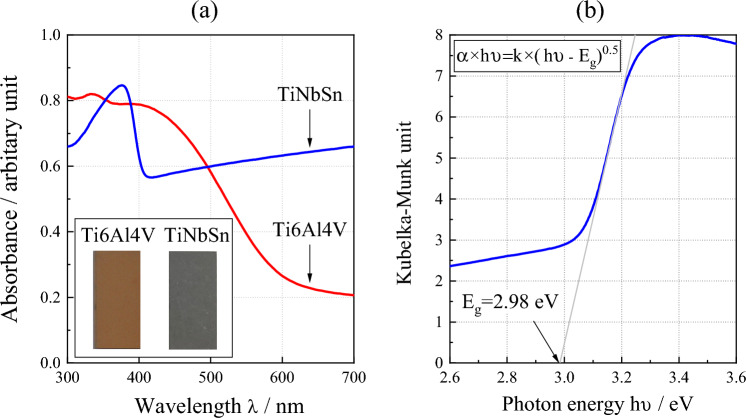
(**a**) Diffused absorption spectra and appearance of the anodic oxides on Ti alloys, and (**b**) plot of the Kubelka–Munk transformation of the absorption spectrum of the oxide on TiNbSn.

**Figure 6:**
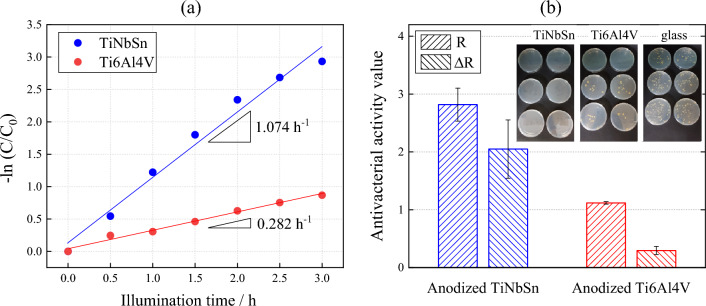
(**a**) Photocatalytic MB decomposition variation of the anodic oxides on the Ti alloys according to UV illumination time, and (**b**) antibacterial activity values and the culture of the *Staphylococcus aureus* washout solution after antibacterial tests of the anodic oxides on the Ti alloys.

**Figure 7:**
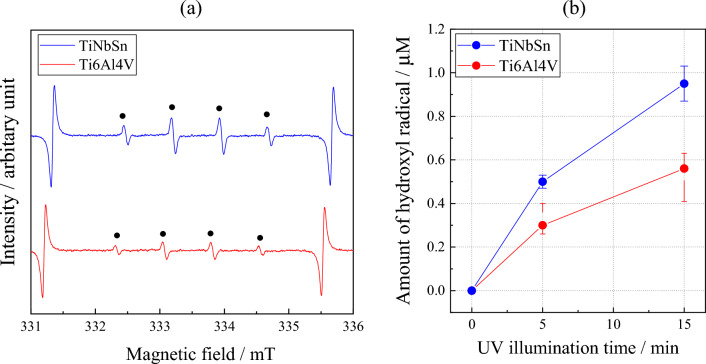
(**a**) DMPO-OH spectra under UV light illumination for 15 min of the anodic oxide on Ti6Al4V and TiNbSn, and (**b**) variation in the average number of ⋅OH (n = 3) radicals with UV light illumination time for the anodic oxides on the Ti alloys.

Figure 5a shows the diffused absorption spectra and appearance of the anodic oxides on Ti6Al4V and TiNbSn. The color of the anodic oxides on Ti6Al4V and TiNbSn is reddish-brown and dark gray, respectively. A band transition is observed at approximately 400 nm for the anodic oxide on TiNbSn; however, a suitable spectrum is not obtained for the anodic oxide on Ti6Al4V. Figure 5b demonstrates a plot of the Kubelka–Munk transformation^38^ of the absorption spectrum of the oxide on TiNbSn. Assuming that the oxide has an allowable direct transition, the bandgap energy was calculated using the Davis Mott equation:

5 $$\alpha \times h\nu = k\times {(h\nu -{E}_{g})}^{n} $$

where α is absorption coefficient; *h* is the Planck's constant; υ is wave number; k is a constant, and *n* is a constant depending on the electron transition under irradiation. The constant *n* = 0.5 was adopted from literature^39^. The bandgap energy of the anodic oxide on TiNbSn is calculated to be 2.98 eV, which is in agreement with the reported value of 3.0 eV^9^. Figure 6 shows photocatalytic properties of the anodized Ti alloys, and Fig. 6a plots the photocatalytic activities of the anodic oxides on Ti6Al4V and TiNbSn against the UV illumination time. The apparent first-order rate constant k_app_ (Eq. 1) for the anodic oxides on the Ti6Al4V and TiNbSn alloys is calculated to be 0.281 and 1.014 h^−1^, respectively, suggesting that the apparent reaction rate of the anodized TiNbSn alloy is approximately 3.6 times higher than that of the anodized Ti6Al4V alloy. The antibacterial activities of the anodized Ti alloys are shown in Fig. 6b. The inset photographs show the culture of the *Staphylococcus aureus* washout solution that was used to rinse the anodized TiNbSn and Ti6Al4V alloys and the glass plate. No colonies are observed for the anodized TiNbSn alloy; conversely, abundant colonies are seen for the anodized Ti6Al4V alloy and glass plate. The antibacterial activity values (Eq. 2) of the anodized TiNbSn are higher than those of the anodized Ti6Al4V. The result implies the strong antibacterial ability of TiO_2_, considering very high levels of antibiotic resistance of that *Staphylococcus aureus*^40^. Figure 7a shows the DMPO-OH spectra of the anodic oxides on Ti alloys under the UV light illumination for 15 min. The variation in the average numbers of ⋅OH (n = 3) with the UV light illumination time is plotted for the anodic oxides on Ti6Al4V and TiNbSn in Fig. 7b. Notably, the number of radicals in the anodized TiNbSn alloy is higher than that in the anodized Ti6Al4V alloy, and the number of hydroxyl radicals increases with illumination time.

## Discussion

The present study identified a strong correlation between the photocatalytic and antibacterial activities of the anodized Ti alloy, suggesting that both properties originate from the same mechanism in which the photogenerated ROS degrade organic substances as well as fungal cells. The activities varied with the substrate; the anodized TiNbSn alloy (rutile TiO_2_) exhibited superior properties to those of the anodized Ti6Al4V (anatase TiO_2_). In general, the photoactivity of the anatase TiO_2_ is superior to that of rutile TiO_2_ because of the longer lifetime of the photogenerated electrons in the anatase than that in the rutile^15^. However, this study suggests that other factors influencing the photoactivity of anodic oxides should also be considered.

The anodization was conducted at a high voltage to prepare highly crystallized TiO_2_, considering that an enhancement in crystallization reduces the lattice defects, which act as recombination sites for the photogenerated charge carriers^37,41^. The lifetime of the photogenerated charge carriers in solids with low defect concentrations is sufficiently long to allow their diffusion to the depletion layer without recombination. Chen et al. reported that higher the crystallinity, fewer the bulk defects, consequently higher the photocatalytic activity^42^. The oxygen vacancies, Ti^3+^ atoms adjacent to O vacancies, interstitial atoms, and interfaces are the probable recombination site defects^41^. The low lattice strain of the anodized TiNbSn alloy is responsible for its high photocatalytic activity. A high voltage during anodization causes Joule heating; consequently, a spark discharge is generated on the electrode surface that leads to the formation of an insulating oxide, thereby resulting in a well-crystallized anodic oxide. Furthermore, a strong electrical field between the electrodes promotes the incorporation of oxygen ions from the solution into the anode, resulting in the formation of oxide pores^43^. Masahashi et al. calculated the amount of excess oxygen based on the valence equilibrium model, which did not participate in oxide formation during anodization, and concluded that the anodic oxide on Ti6Al4V contained approximately 7.3% more excess free oxygen than that on TiNbSn^21^. The high-density pores in the anodic oxide on Ti6Al4V were due to the high amount of free oxygen, and a layered structure consisting of an oxide containing alternating low-density and high-density pores (a mille-feuille structure) is formed. The mechanism of a mille-feuille structure formation is proposed based on the valence equilibrium model^21^. Abundant oxygen ions produced by the oxidation of water molecules by H_2_O_2_ react with trivalent Al in Ti6Al4V, but the excess oxygen ions are not be consumed to form metal oxide. They are either released as gas bulbs on the electrode surface or incorporated into the oxide to form pore enriched layer (Fig. 3a). As a result, a thick anodic oxide with a mille-feuille structure is formed on the Ti6Al4V substrate. Ion transport between the solution and Ti6Al4V electrode facilitated the formation of oxide as well as pores; therefore, the voltage during anodization reduced, and no spark discharge occurred. In contrast, TiNbSn contains a large amount of pentavalent Nb, which binds more oxygen than trivalent Al, thus promoting oxide formation rather than pore formation. The rate of Ti ion transport to the solution interface determines the rate of anodization reaction. Therefore, a higher voltage was required to form an anodic oxide and initiate dielectric breakdown that led to a spark discharge. Consequently, high-temperature-stable rutile TiO_2_ with high crystallinity was formed on the TiNbSn alloy, in contrast to anatase TiO_2_ with low crystallinity on Ti6Al4V. The high crystallinity with few lattice defects, which function as recombination sites, resulted in high photocatalytic and antibacterial activities of the anodic oxide on the TiNbSn alloy.

**Figure 8:**
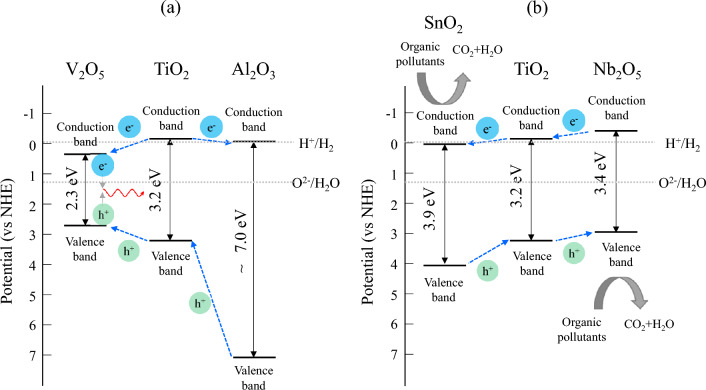
Schematic electronic band diagrams of the heterojunction model for the anodic oxides on (**a**) Ti6Al4V and (**b**) TiNbSn.

Another factor that influences the photocatalytic and antibacterial activities of the anodic oxide on the Ti alloy is the oxides of the alloying element. The present study indicated that the microstructure, crystal structure, absorption behavior, and chemical state of the anodized Ti alloy varied with the substrate, implying that the oxides of the alloying elements in the Ti alloy influenced the quality of the anodic oxide. The calculated fractions of Al_2_O_3_ and Nb_2_O_5_ in the anodic oxides of Ti6Al4V and TiNbSn alloy, respectively, are higher than their estimated fractions (Fig. 4), suggesting their significant contribution in these activities. The bandgap energy of Al_2_O_3_ were studied extensively by experimentalists and theoreticians; however, the metastability of the oxide phase and low degree of crystallinity resulted in a wide range of values: 4.8–5.1 eV^44^, 6.3 eV^45^, 6.6 eV^46^, 2.5–8.7 eV^47^, and 8.8 eV^48^. These high bandgap energies imply that the valence band of Al_2_O_3_ exists at the deep energy position, and the upper edge of the valence band was speculated to facilitate the electron transfer to TiO_2_. The p-type V_2_O_5_ with a bandgap of 2.2 eV^49^ has a lower conduction and an upper valence band edges ~ 0.5 eV higher and ~ 0.4 eV lower, respectively, than those of TiO_2_. Coupling it with n-type TiO_2_ promoted charge separation because of the formation of a depletion zone at the interface between the oxides^50^, and TiO_2_^–^V_2_O_5_ heterojunction semiconductors is expected^51^. In contrast, the n-type Nb_2_O_5_ with a bandgap energy of 3.4 eV had a conduction band edge close to the potential of H^+^/H_2_ and exhibited photocatalytic activity^52^. The conduction band edge of n-type SnO_2_ with a bandgap energy of 3.9 eV was 0.41 eV lower than that of TiO_2_. This was speculated to facilitate interfacial electron transfer, thereby affording the development of heterogeneous photocatalysts to enhance charge separation^53^. Assuming that the individual oxides in the anodic oxide have the same electronic structures as those of their pristine oxides, the schematic band diagrams of the heterojunctions of the oxides on TiNbSn and Ti6Al4V are shown in Fig. 8a, b, respectively. The transfer directions of the photogenerated electrons and holes are shown in the figures. The band structure of the anodic oxides on TiNbSn (Fig. 8a) causes the photogenerated electrons and holes to move in opposite directions due to the positions of valence and conduction band, thereby achieving effective charge separation. Conversely, the photogenerated electrons and holes move toward V_2_O_5_ in the case of anodic oxides on Ti6Al4V (Fig. 8b) because of the highest lower conduction band edge and the lowest upper valence band edge of the V_2_O_5_ among the oxides. This band structure is not favorable for charge separation and prone to recombination. The lifetime of photogenerated charge carriers is a major determinant of the photocatalytic activity because charge-carrier trapping suppresses recombination and prolongs the lifetime^15^. Charge separation across the interface between the constituent substances increased the quantum yield of TiO_2_^54^. This model is based on the heterojunction principle, wherein the charge separation was achieved by co-existing oxides of the alloying elements in the anodic oxides.

The TiNbSn alloy has the lowest Young’s moduli among Ti alloys^55^. Recently, it was approved for manufacturing medical devices by the Ministry of Health, Labor and Welfare, Japan. This study validated that the anodized TiNbSn has photocatalytic and antibacterial activities superior to Ti6Al4V. The photoinduced characteristics of TiO_2_ on the TiNbSn alloy are expected to impart new functions to metallic implant materials and contribute to safe and reliable implant treatment.

## Conclusions

This study revealed the superior photocatalytic and antibacterial activities of the anodized TiNbSn alloy to those of the Ti6Al4V alloy. The results correlated well with the number of hydroxyl radicals generated upon UV light illumination. The anodic oxide on TiNbSn exhibited highly crystallized rutile TiO_2_, whereas that on Ti6Al4V formed poorly crystallized anatase TiO_2_. Self-heating owing to dielectric breakdown with a spark discharge during anodization promoted the crystallinity of the anodic oxide on TiNbSn. In contrast, a spark discharge was not observed on the Ti6Al4V alloy because of the progressive reaction that incorporate oxygen gas to be pores. The oxides of alloying elements appeared in the anodic oxide, and the electronic band structure of the constituent oxides affected the photoexcited charge transfer between the constituent oxides. The high photocatalytic and antibacterial activities of the anodic oxide on the TiNbSn alloy were explained by two mechanisms; the highly crystallized TiO_2_ reduced the recombination of charge carriers and the electronic band structure promoted charge separation through heterojunction formation between the constituent oxides.

## Data Availability

The data that support the findings of this study are available from the corresponding author on reasonable request.
